# Photo-induced optical activity in phase-change memory materials

**DOI:** 10.1038/srep08770

**Published:** 2015-03-05

**Authors:** Konstantin B. Borisenko, Janaki Shanmugam, Benjamin A. O. Williams, Paul Ewart, Behrad Gholipour, Daniel W. Hewak, Rohanah Hussain, Tamás Jávorfi, Giuliano Siligardi, Angus I. Kirkland

**Affiliations:** 1 Department of Materials, University of Oxford, Parks Road, Oxford. OX1 3PH, United Kingdom; 2 Research Complex at Harwell, Rutherford Appleton Laboratory, Harwell Oxford, Didcot OX11 0FA, UK; 3 Clarendon Laboratory, Department of Physics, University of Oxford, Parks Road, Oxford. OX1 3PU, United Kingdom; 4 Optoelectronics Research Centre, University of Southampton, Southampton. SO17 1BJ, United Kingdom; 5 Diamond Light Source, Harwell Science and Innovation Campus, Didcot, Oxfordshire OX11 0DE, United Kingdom

## Abstract

We demonstrate that optical activity in amorphous isotropic thin films of pure Ge_2_Sb_2_Te_5_ and N-doped Ge_2_Sb_2_Te_5_N phase-change memory materials can be induced using rapid photo crystallisation with circularly polarised laser light. The new anisotropic phase transition has been confirmed by circular dichroism measurements. This opens up the possibility of controlled induction of optical activity at the nanosecond time scale for exploitation in a new generation of high-density optical memory, fast chiroptical switches and chiral metamaterials.

Rapid phase-change memory materials, including Ge_2_Sb_2_Te_5_ (GST), are a technologically important class of material used in information storage^1^,^2^,^3^. Information storage in these materials is based on fast (nanosecond) reversible transitions between amorphous and crystalline phases that have different reflectivities and electric conductivities which can be initiated by either an applied electric current^4^,^5^ or by light^6^.

Rapid phase-change memory materials belong to a wider class of chalcogenide alloys which also encompass chalcogenide glasses. In chalcogenide glasses a number of effects have been observed in addition to the primary amorphous/crystalline phase transition when illuminated with light. These include reversible changes that cause a shift of the absorption edge toward longer wavelengths (photo-darkening)^7^,^8^ and changes in refractive index (photo-refraction)^8^,^9^. Related to the polarisation of the incident light, photo-induced anisotropy, which includes photo-reduced linear dichroism and photo-induced linear birefringence, has been observed in chalcogenide films exhibiting photo-darkening and photo-refraction, following exposure to linearly polarized light^10^,^11^,^12^ together with photo-induced gyrotropy^13^. However, all of these effects were observed following long (minute duration) exposure to laser light at wavelengths near the optical bandgap.

These effects are not limited to chalcogenide alloys. In other materials, photo-induced optical anisotropy was first observed in AgCl illuminated by polarised light by Weigert^14^. It has been demonstrated that optical activity can be induced using circularly polarised light in achiral polymers^15^,^16^. However, as with chalcogenide glasses, these changes require long (minute duration) illumination times.

Whilst these examples show that optical activity in principle can be induced in otherwise achiral media, optical activity in inorganic materials induced using circularly polarised light on nanosecond time scales has not been demonstrated before.

The control of photo-induced properties in chalcogenide phase-change memory materials is technologically important for information storage where optical anisotropy can be used as an additional information storage parameter. More generally, the fast chirality induction can be exploited in chiroptical switches^17^. Chiral semiconductors may have applications as chiral metamaterials^18^ or chiral sensors^19^,^20^.

In this work we show that optical activity together with a phase change can be induced in thin amorphous films of pure and doped GST using short pulses of circularly polarised light. The handedness of the light used to induce crystallisation produces a mirror-image circular dichroism (CD) response in certain spectral regions. These suggest that control of anisotropic optical properties in these and related materials using polarised light is possible and that the direction of illumination polarisation can be recorded and therefore used as an additional parameter for information storage.

## Results

**Figure 1:**
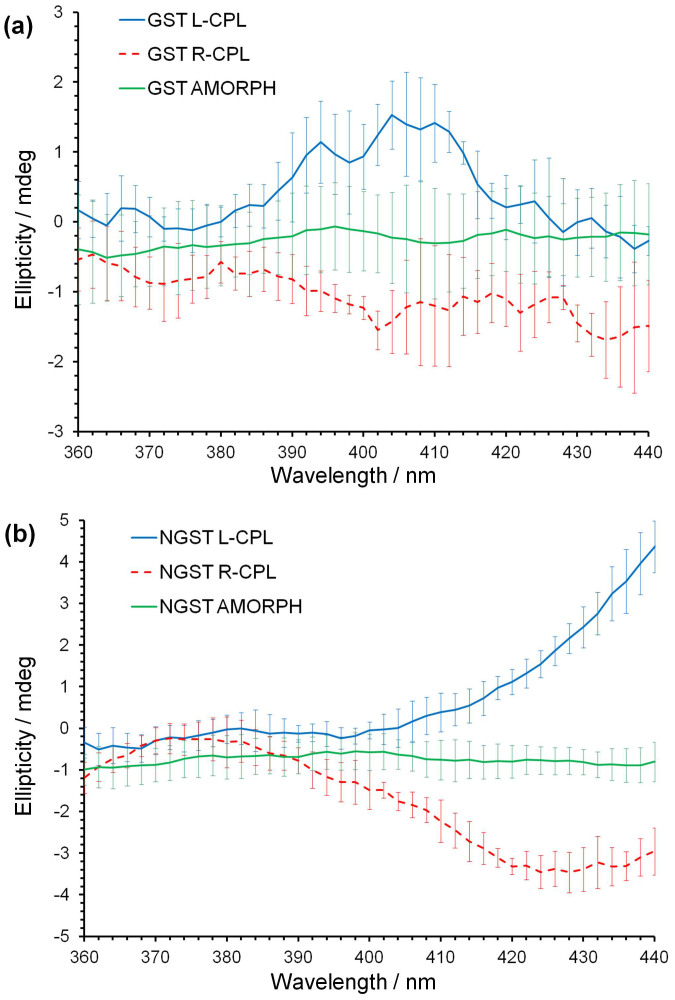
CD spectra from pure and N-doped GST films. (a) Single CD spectra of pure GST thin film crystallised by pulses of L-CPL and R-CPL together with averaged spectrum from amorphous regions (GST AMORPH). Error bars show standard deviations from five repeated measurements. (b) Double CD spectra of N-doped GST thin film crystallised by pulses of L-CPL and R-CPL together with the averaged double CD spectrum from amorphous regions (NGST AMORPH). Error bars show standard deviations from five repeated measurements.

CD spectra from both amorphous and crystallised regions of pure and N-doped GST induced by left (L) and right (R) circularly polarised light (CPL) are shown in Figure 1.

There is a clear difference between the CD spectra from crystallised areas induced by left and right circularly polarised light in the wavelength range from 380 to 420 nm for pure GST (Figure 1a) and from 410 to 440 nm for NGST (Figure 1b). This difference between the L-CPL and R-CPL spectra is larger for NGST and for both GST and NGST films, spectra recorded from an area crystallised by L-CPL have consistently higher CD values than those recorded from an area crystallised by R-CPL.

Importantly, spectra recorded from crystallised NGST show pronounced mirror symmetry dependent on the handedness of light used to induce crystallisation when compared to spectra recorded from amorphous NGST in the wavelength range between 410–440 nm (Figure 1b). A similar trend within the same wavelength range is also observed for pure GST films, although the changes between spectra recorded from crystallised and amorphous states are much smaller and generally lie within experimental standard deviations (Figure 1a).

## Discussion

Statistically significant differences between the CD spectra obtained from crystallised areas of NGST films using different handedness of light demonstrate successful differentiation between the two states of material which can be used to represent two bits of information stored in the same volume.

In general, circular dichroism arises from the difference in absorbance between left- and right-circularly polarized light passing through the sample and a CD signal can be obtained only from objects or structural motifs that are chiral. For this reason we propose that there are several possible mechanisms for the observed induced CD effects.

**Figure 2:**
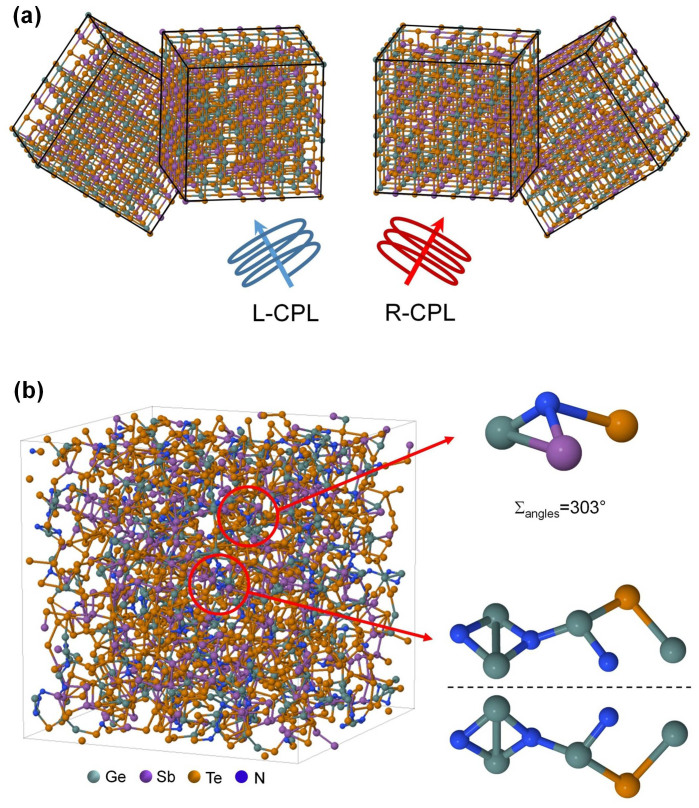
Proposed mechanisms of the induced CD signal. (a) A schematical illustration of alignment of individual crystallites along polarisation vector as circularly polarised light propagates through the material. The illustrated crystallites form a chiral crystallite cluster thought responsible for the observed CD signal. (b) Chiral atomic structural motifs proposed to contribute to the observed chiral signal in NGST.

One explanation is that the electric field of the light used to induce crystallisation influences the direction of crystallisation. In this mechanism the growing crystallites align along the rotating polarisation vector of the incident light, forming a chiral structure from crystallites of the material as the light propagates through the film, as schematically demonstrated in Figure 2a.

Previously polarisation-induced anisotropy of crystallite growth was observed in amorphous Se^21^,^22^,^23^ and Se-based Se_70_Ag_15_I_15_ chalcogenide films^24^ during photo crystallisation. It has been proposed that photo excitation is important for the observed anisotropy, although an alternative mechanism has been suggested based on purely thermal effects that produce similar alignment due to differential absorption of polarised light by differently oriented crystallites^23^.

Although exact mechanism of such alignment will require further investigation, it seems reasonable that similar mechanisms may be at work in the present case. Previously photo excitation and electronic effects below melting point were found to be important for phase transition in phase-change memory materials^25^,^26^,^27^,^28^. In addition, electric fields have been suggested to influence crystallisation not only in chalcogenides^29^,^30^,^31^ but in other materials, such as silicon^32^.

An alternative, or possibly additional, mechanism is based on the formation of an excess of chiral defects within and at the surface of the crystallites formed or enhanced by chiral illumination. It has been suggested that interaction with polarised light results in alignment of charged defects in a chalcogenide glass^33^. Although such charged defects have never been observed in phase-change memory materials, it seems reasonable to speculate the possibility of such defects based on the similarities of the composition of chalcogenide glasses and phase-change memory materials. Optically active defects can form in GST as a consequence of a previously suggested mechanism for crystallisation/amorphisation involving rotations of nanoscale layers of crystalline lattice possibly present in amorphous phase^34^ to form crystallites. Presence of nanoscale or medium range order in amorphous phase of chalcogenide phase-change memory materials has been suggested as a reason for the observed fast crystallisation of these materials^34^,^35^. In support of the formation of chiral fragments in GST, helical chains of Ge atoms were suggested to be responsible for the observed mirror-symmetric magneto-optical Kerr rotation in layered [(GeTe)_2_(Sb_2_Te_3_)_1_]_n_ films^36^. Suitably chiral GeN clusters can be discerned in the refined atomic structure of NGST from previous electron diffraction and density functional theory study (Figure 2b) together with pyramidal nitrogen bonded to three different groups^37^. The presence of these is consistent with the enhanced CD signal observed in the NGST films.

The experimentally observed increase in CD signal for NGST compared to pure GST may be also related to a lower optical absorption which required measurement from a thicker film of the former, 45 nm, as compared to 30 nm for GST film. In this situation the degree of alignment of crystallites along the light propagation axis as well as the amount of the chiral centres can be enhanced for a thicker film as a result of a longer light-matter interaction during crystallisation.

We have shown that the amorphous to crystalline phase-transition in both pure Ge_2_Sb_2_Te_5_ and N-doped Ge_2_Sb_2_Te_5_N chalcogenides can be successfully induced by nanosecond laser pulses with left- and right-circularly polarised light resulting in optically active material. This has been experimentally demonstrated from measurements of CD spectra from small crystallised areas of thin films of both materials using the unique capabilities of beamline B23 at Diamond Light Source. Importantly, the high photon flux in a highly collimated beam with a small cross-section available at this beamline has enabled the measurements of the CD spectra from small areas of both GST and NGST using a novel technique that doubles CD signal similar to that for magnetic CD (MCD) measurements.

The CD spectra recorded from materials crystallised using left or right-handed CPL show notable differences and pronounced mirror symmetry in changes in the spectra between amorphous to crystalline regions of the samples. In the case of pure GST the changes between amorphous and crystalline spectra are within or close to experimental errors but are considerably more pronounced in the spectra of NGST with a consistent sign for both materials.

This ability to rapidly induce optical activity in GST and NGST films during a phase transition opens up new potential for increased information storage capacity using these materials. More generally, current observations show that chirality control using circularly polarized light is feasible in thin films of inorganic materials at nanosecond time scale with a potential for control of anisotropic optical properties for optoelectronic and photonics applications.

## Methods

### Thin film growth

Pure Ge_2_Sb_2_Te_5_ and 10 at % N-doped Ge_2_Sb_2_Te_5_N amorphous films were deposited by magnetron sputtering on 20 mm diameter 1.25 mm thick fused silica glass disk substrates. The 10 at % N doping was achieved using plasma nitridation during film deposition. The deposited film thicknesses were selected to give an optical absorption in the films of approximately 1 between wavelengths of 300–200 nm after the phase transition was induced. These required film thicknesses were established as approximately 30 and 45 nm for pure and N-doped GST, respectively. For all samples a 20 nm capping layer of silica was deposited to protect the films from oxidation.

### Phase transformation induction

Phase changes in the as-prepared films were induced using pulses of left or right CPL generated using a Nd:YAG laser at wavelengths of 532 nm (second harmonic). The desired polarisation state was generated using a combination of a linear polarizer and a suitable quarter waveplate. The linear polarizer (a Glan-Taylor prism) was used first to ensure a 100% polarization state at the input to the quarter waveplate. The laser beam was subsequently switched between left and right-handed circularly polarised states by rotating the waveplate to the corresponding angles. The final size of the laser spot on the sample disk was controlled by a masking aperture and was approximately 2 mm in diameter. Typically ten pulses with durations between 7 and 8 ns (FWHM) each and a per-pulse energy of 0.3(1) mJ were used to induce crystallization in the films. The experimental optical setup used is shown in the Supplementary Material (Figure S1).

### CD spectra measurements

**Figure 3:**
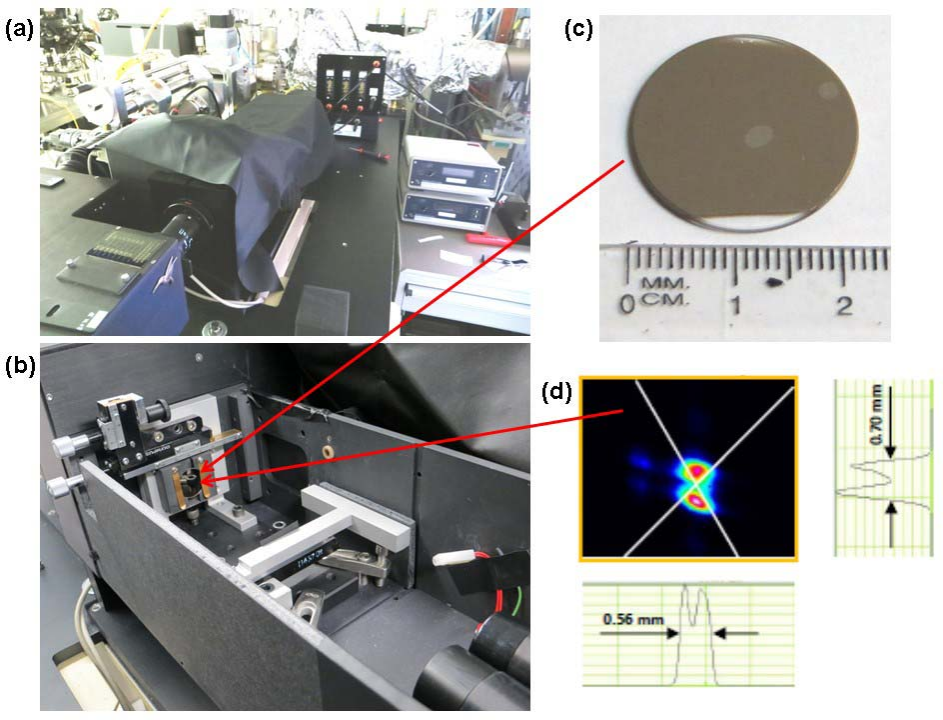
Measurements of the CD spectra. (a) Sample chamber at circular dichroism beamline B23 module B. (b) Sample chamber without the lid showing the holder that allows vertical and horizontal movement of the sample. (c) Fused silica disk sample coated with a thin film of GST showing two crystallised areas (the crystallised area in the centre of the sample was used for measurements of the CD spectra). (d) Beam profile of the beam at B23 module B measured at the sample plane using the beam profile monitor Beamage USB.

CD spectra from a crystallised area approximately 2 mm in diameter in both GST and NGST films were measured using the highly collimated incident microlight beam generated by circular dichroism beamline B23 at Diamond Light Source at CD module station B (Figure 3)^38^,^39^. Measurements of CD spectra from such small areas are not possible using bench-top CD instruments with light beam cross section of about 80 mm^2^ (8 mm × 10 mm). The magnification of the optical arrangement used is 1:1 and the diameter of the incident light beam at the beamline is about 0.6 mm enabling the beam to be positioned at two different locations within the crystallized area on the sample. CD spectra were recorded in the UV-visible spectral range between 180 and 500 nm with a 2 nm step using 2 nm bandwidth. Importantly, the high photon flux of the beamline^38^ permitted sufficient transmission of light through the sample films even after crystallisation.

A customised vertical mounting stage for the sample holder installed in the sample chamber of the beamline (Figures 3a and 3b) enabled horizontal and vertical positioning and measurements of CD spectra in different areas of the sample disk (Figure 3c). All measurements reported included two different positions within each crystallised spot in addition to two to three different positions within each of the as-prepared amorphous films. Spectra from the amorphous regions served as references that were used to gauge the changes in the CD spectra from the crystallised regions.

To assess the effects of linear dichroism in the observed CD spectra from the crystallised regions of the samples studied, a Rochon prism (polarising beam splitter) was inserted in the beamline before the photoelastic modulator. This prism splits the incident beam into two orthogonally linearly polarised beams. Hence rotation of the Rochon prism between two orthogonal positions has the effect of rotating the incident light polarisation on the sample by 90 degrees. This is equivalent to rotation of the sample relative to the incident beam but with the important advantage that the spectrum is recorded from exactly the same sample area. Recording CD spectra at two positions of the Rochon prism therefore provides an estimate of the contribution of linear effects by summation of the two spectra. In the absence of any linear dichroism signal from the crystallised area the sum of the two spectra should be equal to the sum of two similar spectra recorded from the achiral amorphous regions. Consequently, the difference between the two spectra obtained with different rotation of the Rochon prism is effectively a double CD signal from the material. In this study five repeated scans were recorded for each measurement and each sample was measured using two orthogonal polarisations of the incident light resulting from two different rotations of the Rochon prism. Spectra averaged over repeated measurements for each Rochon prism position recorded from amorphous regions of the samples were used as reference in the final analysis. Spectra from crystallised regions of the sample showing maximum absorption, implying maximal degree of crystallisation were selected for comparison with the spectra from the amorphous regions. The errors in the spectra were estimated as a standard deviation computed from repeated collections of the spectra.

The final spectra were examined in the spectral range between 360–440 nm (in the region of the operational wavelength of a semiconductor Blue-Ray^tm^ laser at *ca*. 400 nm) for both films. A more detailed description of the analysis of individual experimental data is given in the Supplementary Material.

## Author Contributions

K.B.B. conceived the study, performed the CD spectral measurements, and wrote the paper. J.S. helped with the CD spectral measurements and data analysis and performed the SEM work. A.I.K. helped with data analysis and contributed to writing the paper. B.A.O.W. and P.E. induced photo crystallisation. B.G. and D.W.H. helped with initial stages of the phase transformation induction. T.J., R.H. and G.S. developed the CD measurement technique. All authors discussed the results.

## Supplementary Material

Supplementary Information Supplementary Material
