# Supplementary material for: Photo-induced optical activity in phase-change memory materials
Source: Sci Rep. 2015 Mar 5;5:8770. doi: 10.1038/srep08770 (PMC4350098; doi:10.1038/srep08770)
Supplement: Supplementary Information — Supplementary Material [file srep08770-s1.pdf]

Photo-induced optical activity in phase-change memory materials

Konstantin B. Borisenko[1,2,*], Janaki Shanmugam[1,2], Benjamin A. O. Williams[3], Paul Ewart[3], Behrad Gholipour[4], Daniel W. Hewak[4], Rohanah Hussain[5], Tamás Jávorfi[5], Giuliano Siligardi[5] and Angus I. Kirkland[1,2]

[1] *Department of Materials, University of Oxford, Parks Road, Oxford, OX1 3PH, United Kingdom*

[2] *Research Complex at Harwell, Rutherford Appleton Laboratory, Harwell Oxford, Didcot OX11 0FA, UK*

[3] *Clarendon Laboratory, Department of Physics, University of Oxford, Parks Road, Oxford, OX1 3PU, United Kingdom*

[4] *Optoelectronics Research Centre, University of Southampton, Southampton, SO17 1BJ, United Kingdom*

[5] *Diamond Light Source, Harwell Science and Innovation Campus, Didcot, Oxfordshire OX11 0DE, United Kingdom*

* Corresponding author. E-mail: konstantin.borisenko@materials.ox.ac.uk,

Supplementary material

Photo-induced crystallisation

The experimental optical setup for the photo-induced crystallisation is shown in Figure S1.

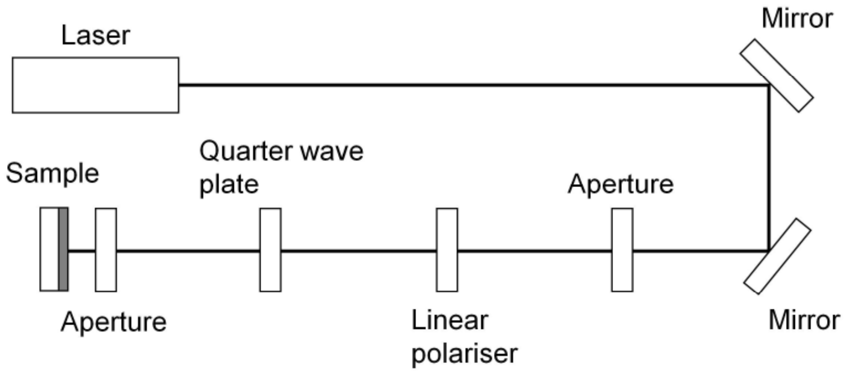

Figure S1. Experimental optical setup for photo-induced crystallisation of GST and NGST films.

Crystallised area analysis

The crystallised areas on the amorphous samples were examined by optical microscopy (Figure S2) and scanning electron microscopy (SEM). The SEM images show that the crystallised spots on the GST and NGST samples are not uniform, although the uniformity is higher in the NGST films (Figures S3, S4). For pure GST samples areas where the irradiation by laser light has caused damage to the capping layer are also observed, appearing as dendritic structures (Figure S4(d).)

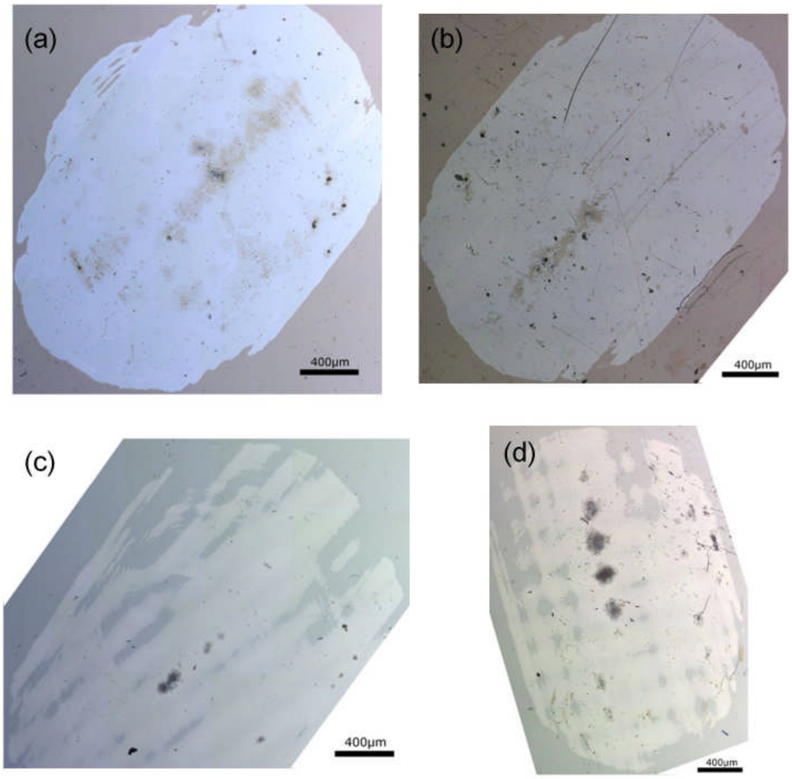

Figure S2. Optical images of laser-irradiated surfaces of GST and NGST films (a) GST film irradiated by right circularly polarized light (RCPL), (b) GST film irradiated by left circularly polarized light (LCPL), (c) NGST irradiated by RCPL, (d) NGST irradiated by LCPL.

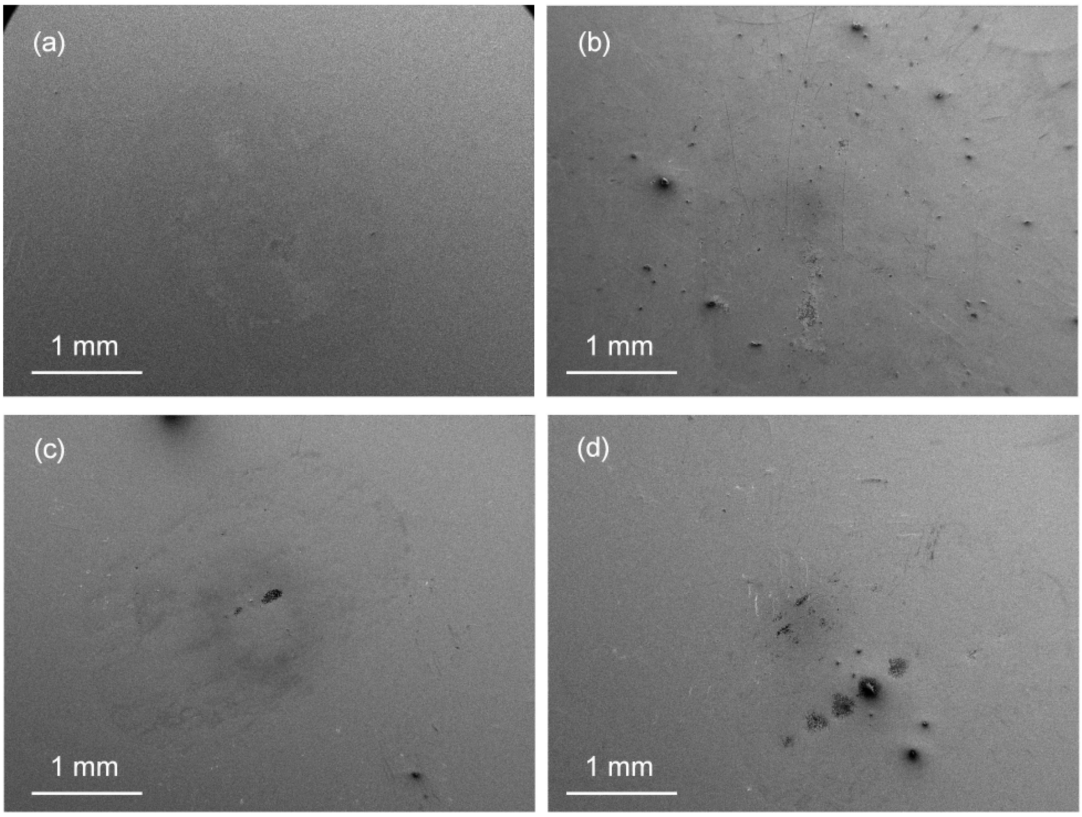

Figure S3. SEM micrographs of laser-irradiated surfaces of GST and NGST films. (a) GST film irradiated by right circularly polarized light (RCPL), (b) GST film irradiated by left circularly polarized light (LCPL), (c) NGST irradiated by RCPL, (d) NGST irradiated by LCPL.

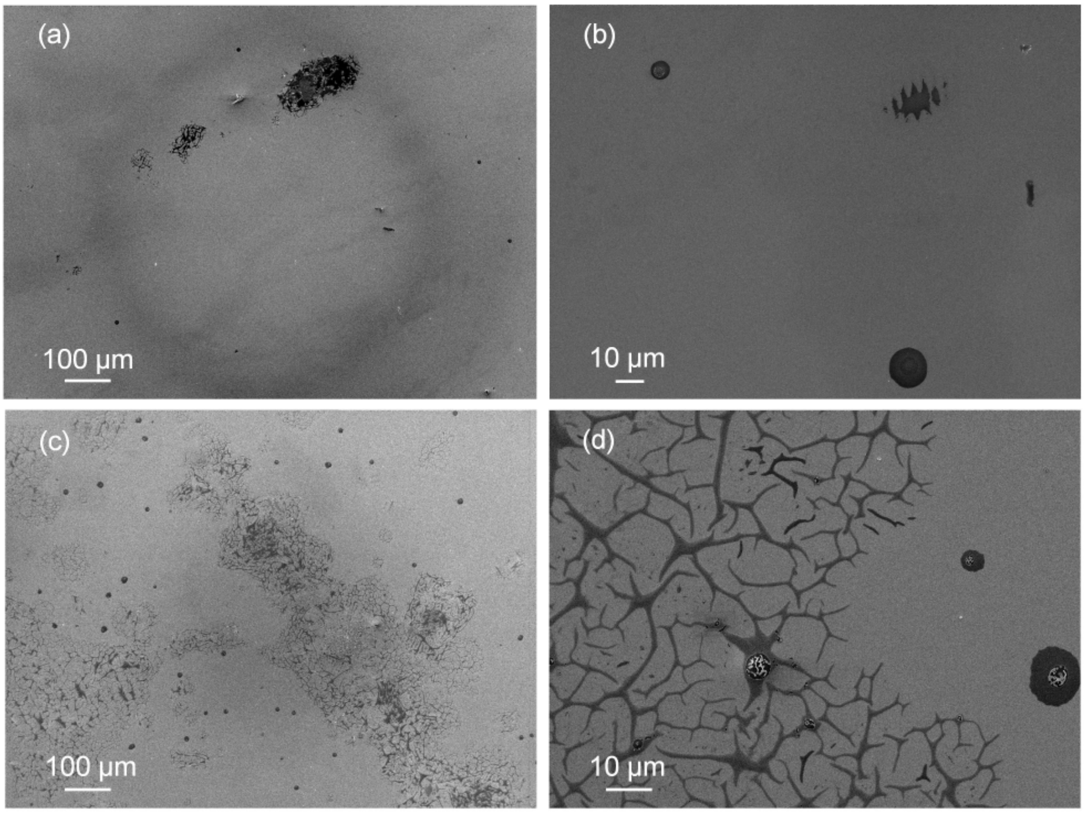

Figure S4. SEM micrographs of (a,b) laser-irradiated NGST film surfaces showing uniform texture (a,b). Laser-irradiated GST film surfaces with dendrite-like features and dark spots (c,d).

CD spectra analysis

CD spectra were measured with two positions of rotation of the Rochon prism, denoted as R1 and R2 (differing by 90º), introduced in the optical path before the photoelastic modulator. The CD spectra from different amorphous regions of pure and N-doped GST films are all similar (Figures S5-S8), as would be expected from the achiral as-prepared amorphous films.

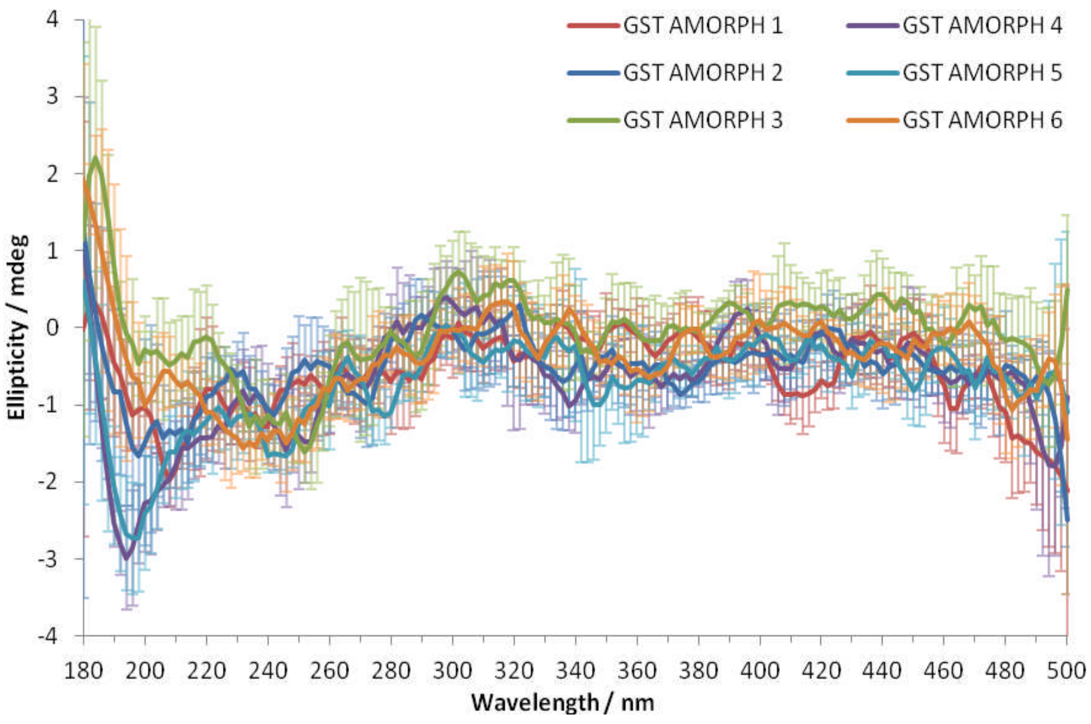

Figure S5. Individual spectra collected from three different amorphous regions on two GST films samples (GST AMORPH 1,2,3 and GST AMORPH 4,5,6) at the R1 prism position.

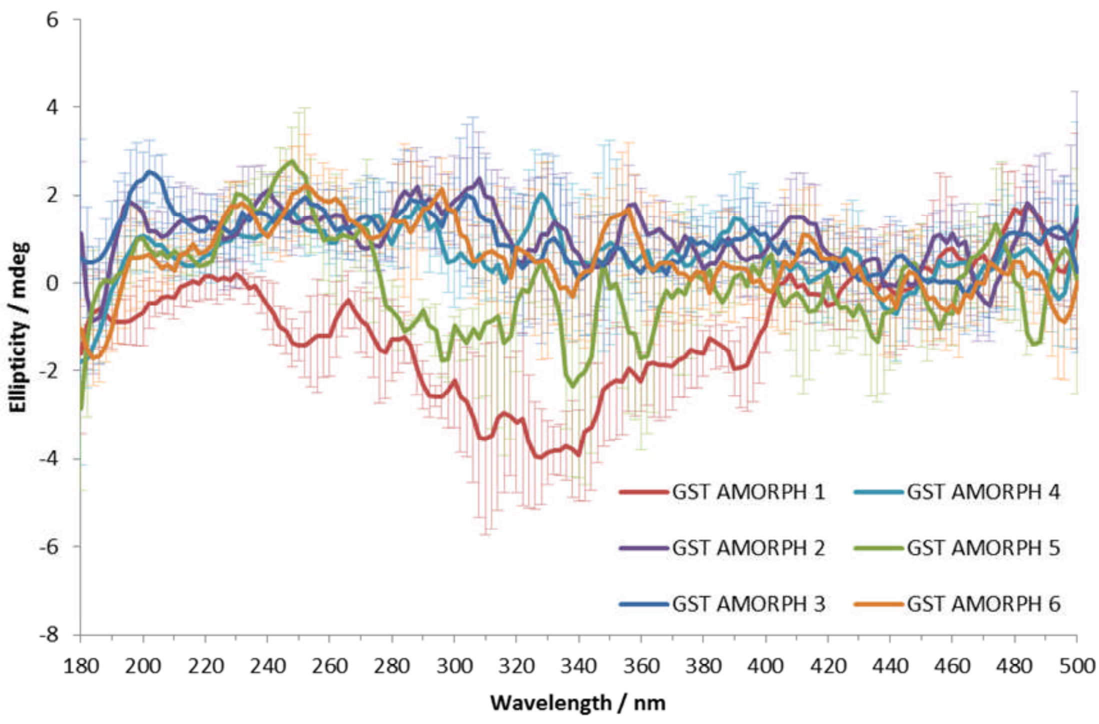

Figure S6. Individual spectra collected from three different amorphous regions on two GST films samples (GST AMORPH 1,2,3 and GST AMORPH 4,5,6) at the R2 prism position.

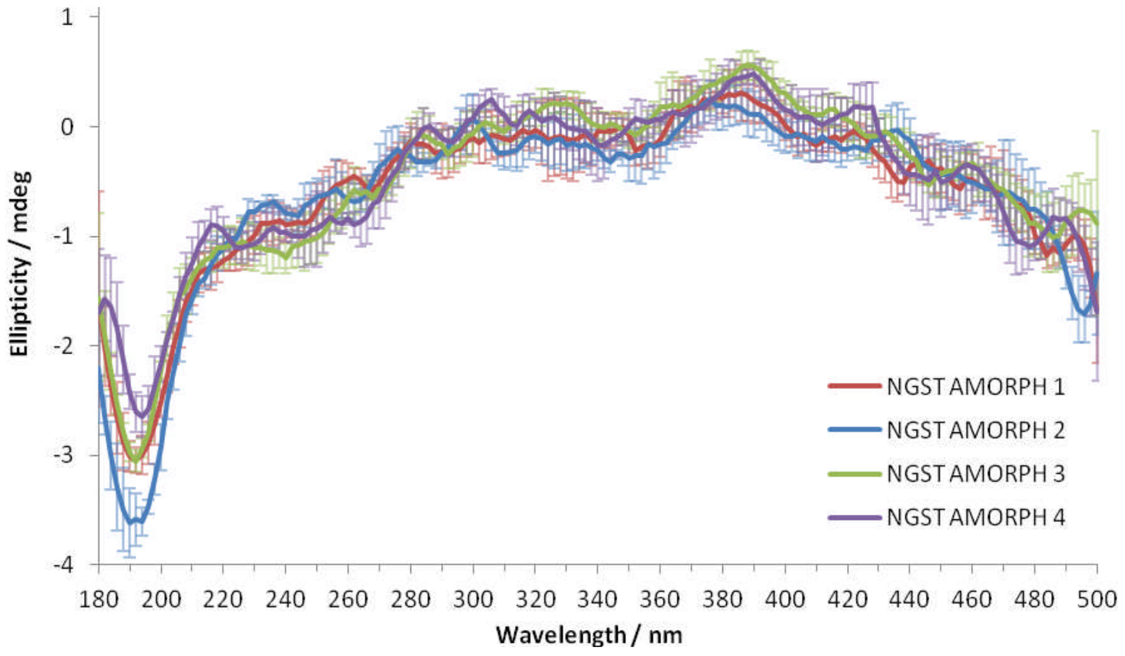

Figure S7. Individual spectra collected from two different amorphous regions on two NGST films samples (NGST AMORPH 1,2 and NGST AMORPH 3,4) at the R1 prism position.

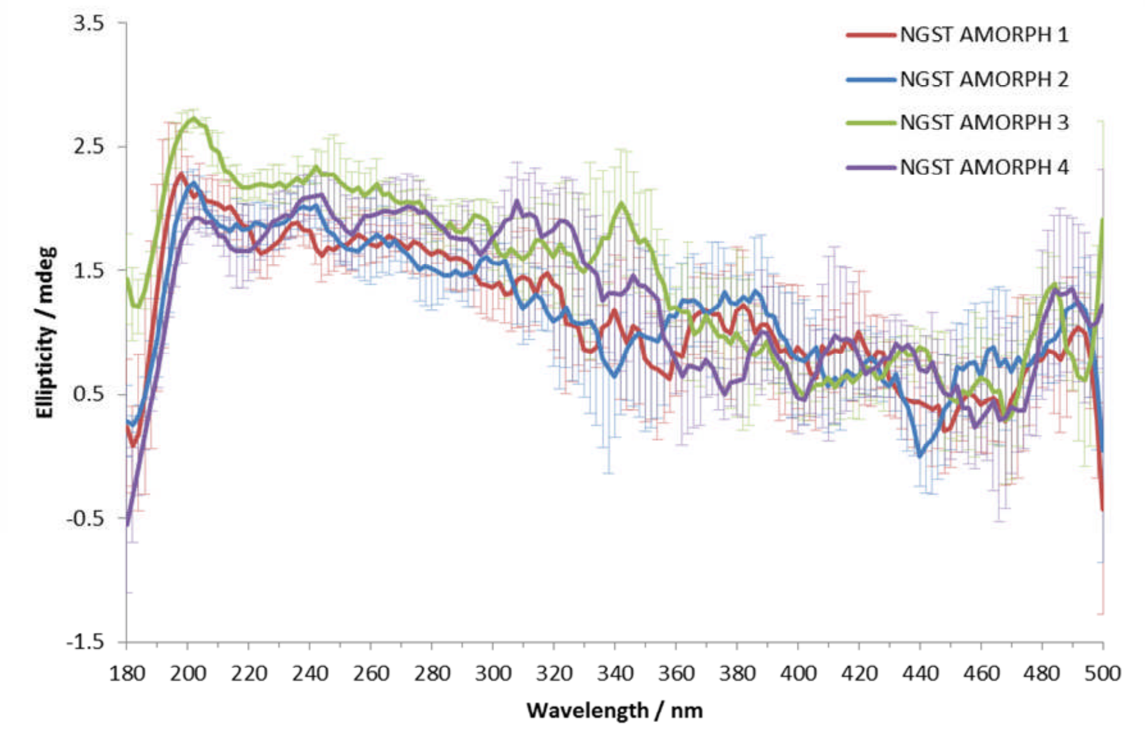

Figure S8. Individual spectra collected from two different amorphous regions on two NGST films samples (NGST AMORPH 1,2 and NGST AMORPH 3,4) at the R2 prism position.

Elliptical polarisation of the B23 beam profile and alignment of the measurement equipment resulted in the beam intensity at the sample varying with rotation of the Rochon prism. The highest beam intensity is achieved at the R1 position whereas the R2 position results in a lower beam intensity. Consequently this leads to lower transmitted beam intensity and higher noise levels in the CD spectra at the position R2 and further implies that the sample film thickness and its corresponding optical transmission play an important part in measurements of the spectra. As the absorption of pure GST is higher than that of NGST, the spectra of GST recorded at the R2 position of the prism have higher levels of noise. The lower intensity beam at the sample may also result in spectral artefacts for more strongly absorbing GST films, when the photomultiplier (PMT) voltage approaches 700 V, its saturation limit. Hence, spectra obtained at the R2 rotation of the prism are excluded from our analysis of the pure GST CD spectra; and only single CD spectra (recorded at the R1 prism position) are examined for these films.

The implementation of a rotating Rochon prism allows the weak CD signal to be effectively doubled by taking a difference between the spectra recorded for different prism rotations. In this case, errors obtained in the individual spectra are propagated to the difference in the calculations of the final spectra. For NGST films, the difference between R1 and R2 spectra was also used to analyse the double CD signal.

For an ideal case, the sum of the spectra recorded at different prism positions should be zero and any deviations from zero indicates a contribution from linear dichroism in the sample film, sample substrate or contributions from the instrumental background. Therefore, the sum (R1+R2) can also be used to estimate the contribution to the signal due to linear dichroism. The square root of the sum of

squared deviations of the averaged summed spectra recorded from crystallised areas subtracted from averaged summed spectra recorded from amorphous areas represents the deviation from the ideal background (which is the amorphous spectrum) due to contributions from the linear dichroism of the sample and instrumental background. This is plotted in Figures S12, S13 for the two GST and NGST films studied. Analysis of this data indicates that between wavelengths from 210 to 490 nm for NGST and between wavelengths from 210 to 450 nm for GST the contribution from linear dichroism is less than the experimental error (computed as twice standard deviation). This implies that contribution from the linear dichroism is negligible in this wavelength range.

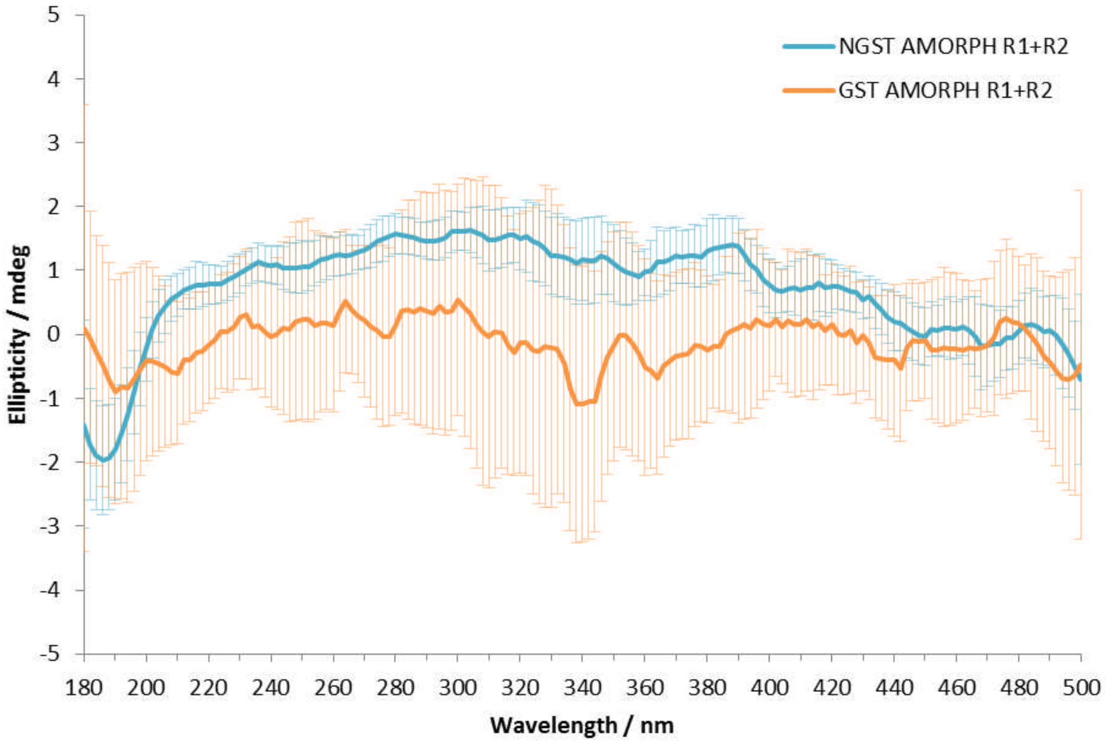

Figure S9. Comparison of the sums of the averaged spectra recorded at R1 and R2 prism positions of amorphous regions on GST (GST R1+R2) and NGST (NGST R1+R2) films.

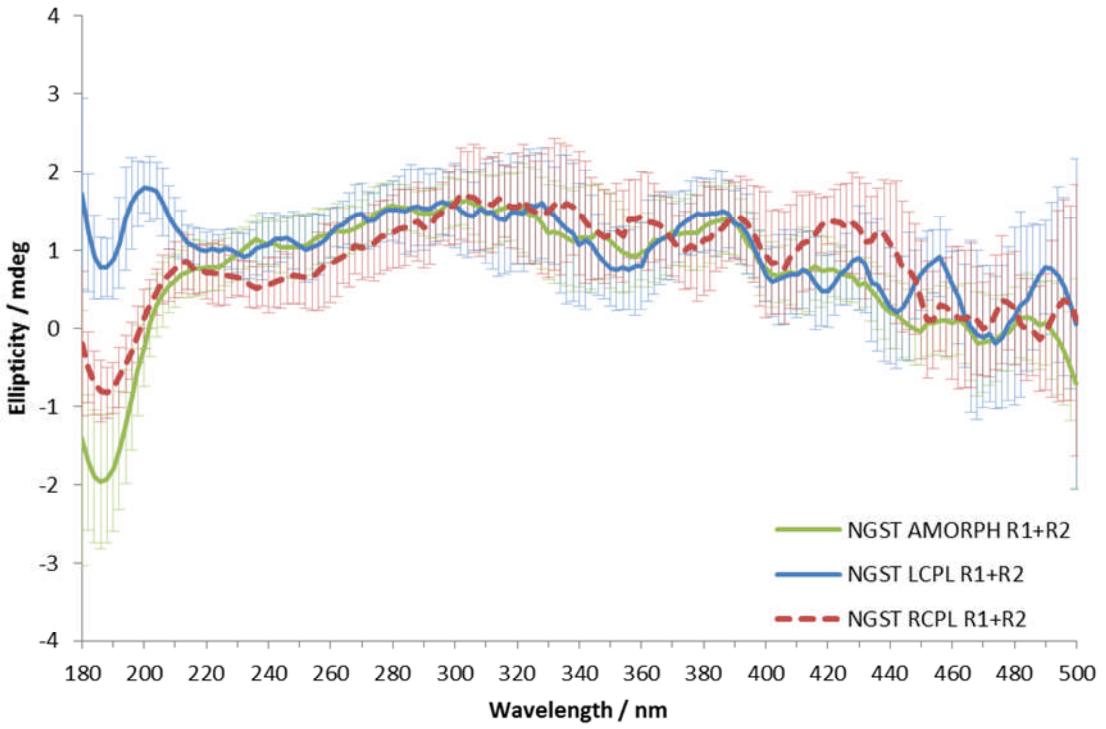

Figure S10. Comparison of summed average spectra of amorphous regions (NGST AMORPH R1+R2) and regions crystallised using LCPL (NGST LCPL R1+R2) and RCPL (NGST LCPL R1+R2) laser light on NGST films at R1 and R2 prism positions.

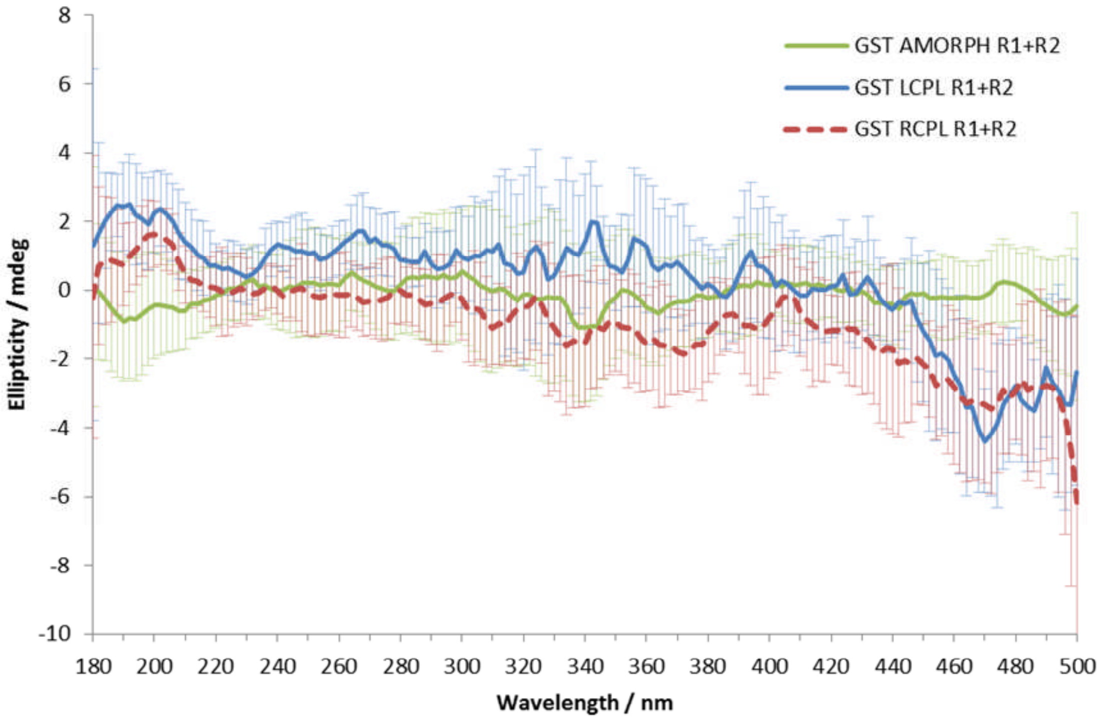

Figure S11. Comparison of summed average spectra of amorphous regions (GST AMORPH R1+R2) and regions crystallised using LCPL (GST LCPL R1+R2) and RCPL (GST LCPL R1+R2) laser light on GST films at R1 and R2 prism positions.

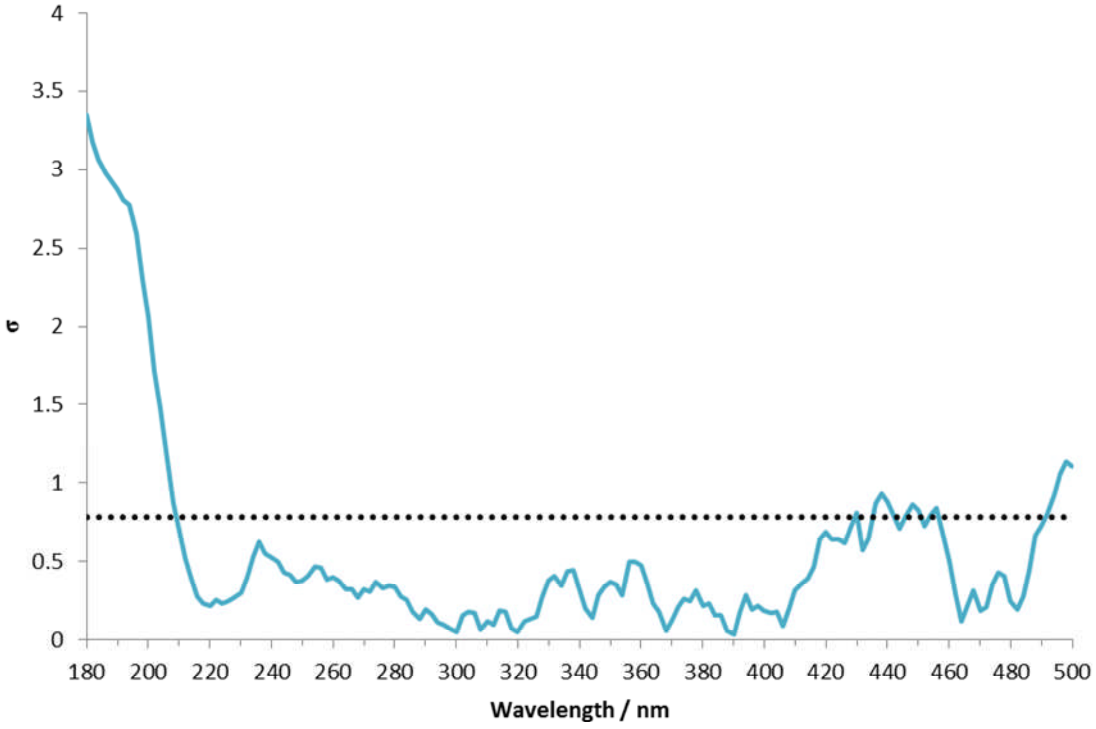

Figure S12. Absolute deviation of averaged R1+R2 spectra recorded from crystallised areas subtracted from averaged R1+R2 amorphous spectrum for NGST film. Averaged error from the spectra of crystallised areas is shown as a dotted line. Large deviation above the dotted line indicates spectral region with possible contribution from linear dichroism to the CD spectra.

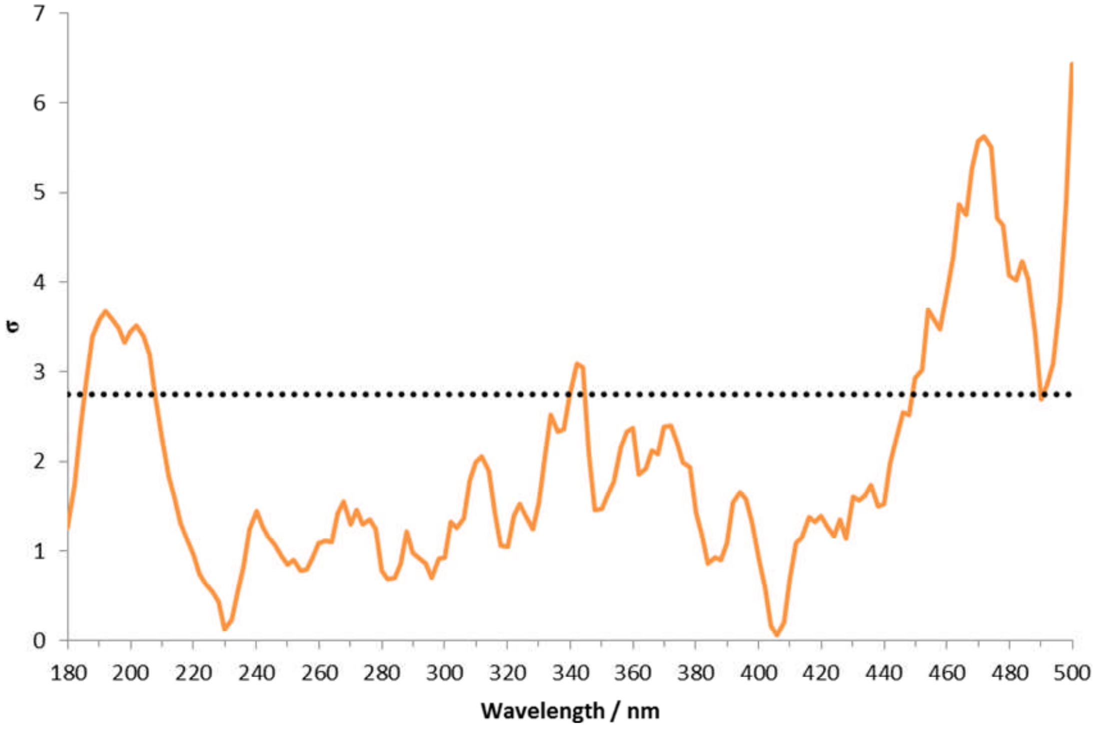

Figure S13. Absolute deviation of averaged R1+R2 spectra recorded from crystallised areas subtracted form averaged R1+R2 amorphous spectrum for GST film. Averaged error from the spectra of crystallised areas is shown as a dotted line.

Large deviation above the dotted line indicates spectral region with possible contribution from linear dichroism to the CD spectra.

The detector photomultiplier (PMT) voltages are proportional to the optical absorption and can be used as a measure of the position of the probe spot on the sample. As the crystallised spots are relatively small and not completely uniform, the higher PMT voltages indicate larger absorption and correspond to the positioning of the probe spot on the most extensively crystallised region. For the analysis described below the spectra from the crystallised areas which have the highest PMT voltage (higher absorption) values for the R1 prism position were selected, which gives the most intensive beam on the sample. The corresponding PMT voltage variations with wavelength for all measured spectra are shown in Figures S14-S21.

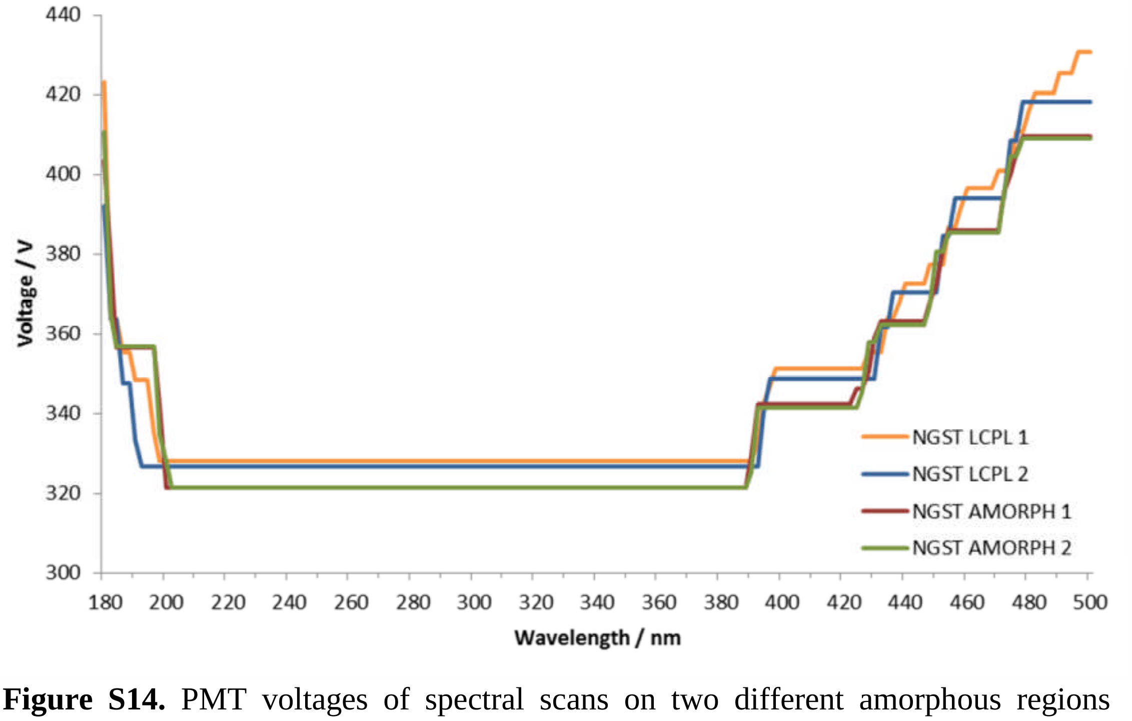

Figure S14. PMT voltages of spectral scans on two different amorphous regions (NGST AMORPH 1,2) and two different positions within the crystallised area using the LCPL laser light (NGST LCPL 1,2) at R1 prism position for NGST film sample.

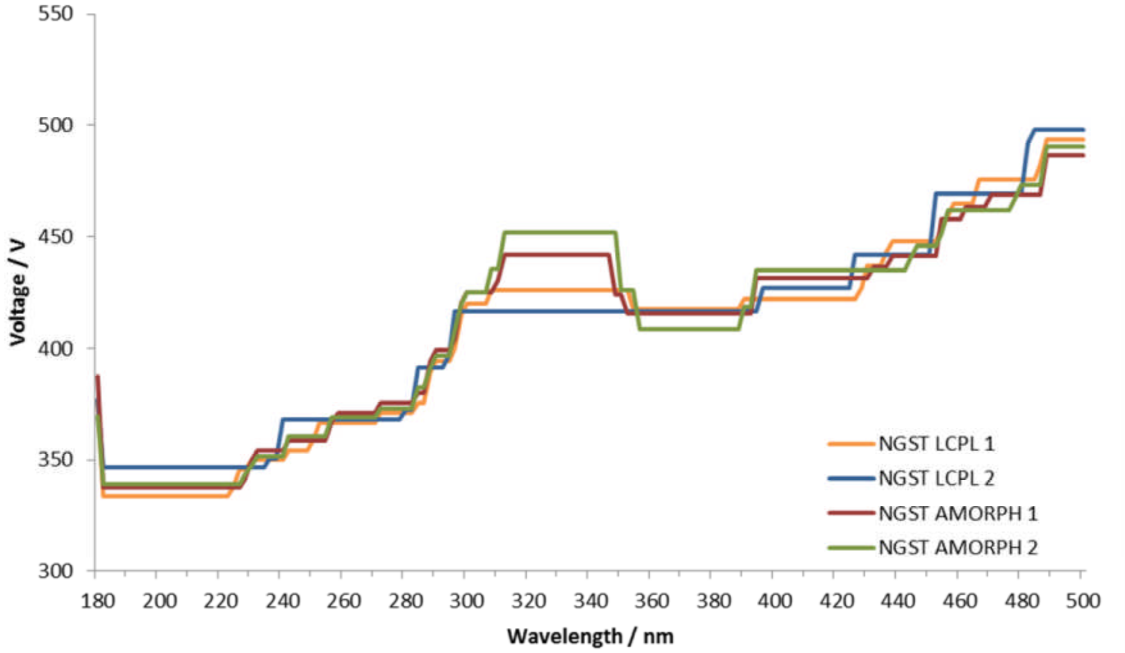

Figure S15 PMT voltages of spectral scans on two different amorphous regions (NGST AMORPH 1,2) and two different positions within the crystallised area using the LCPL laser light (NGST LCPL 1,2) at R2 prism position for NGST film sample.

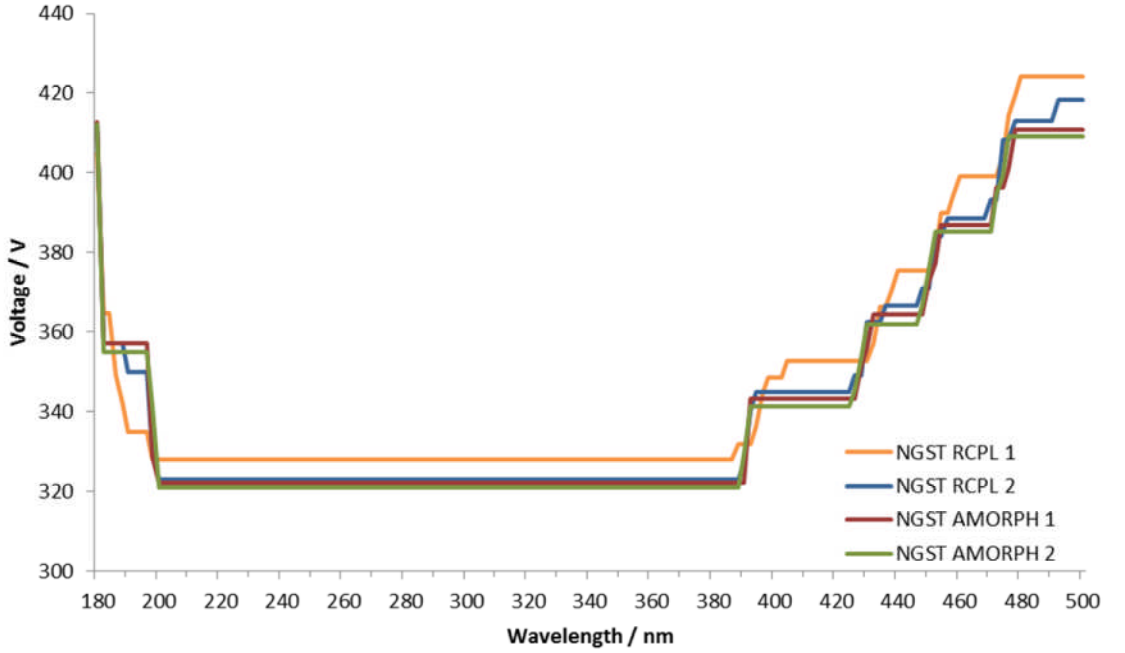

Figure S16. PMT voltages of spectral scans on two different amorphous regions (NGST AMORPH 1,2) and two different positions within the crystallised area using the RCPL laser light (NGST RCPL 1,2) at R1 prism position for NGST film sample.

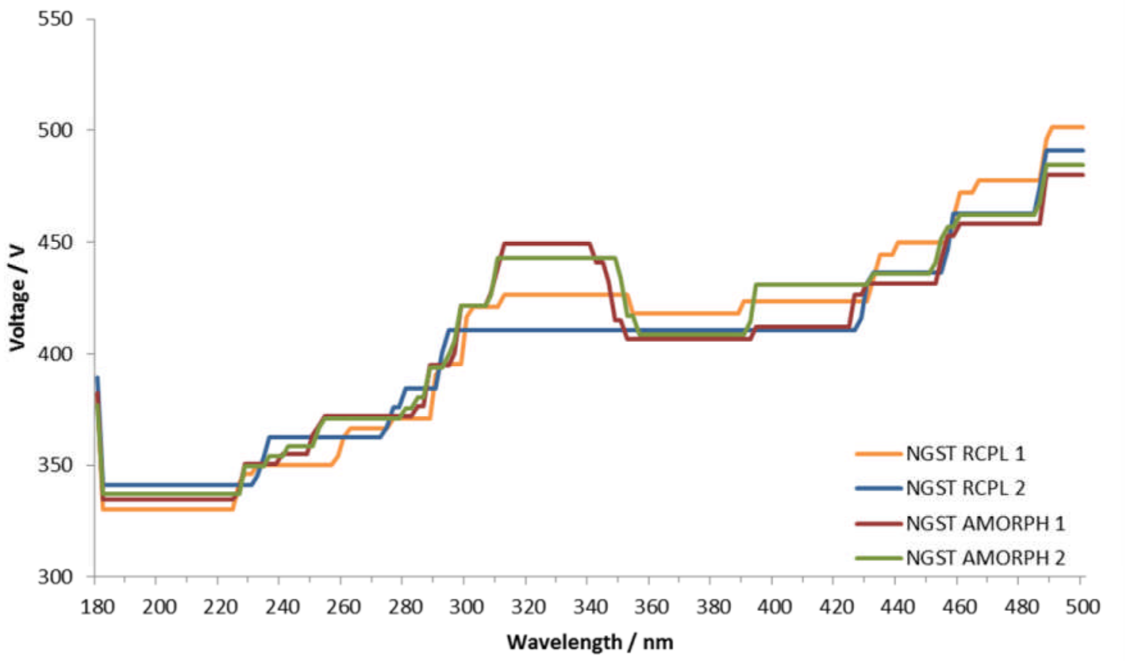

Figure S17. PMT voltages of spectral scans on two different amorphous regions (NGST AMORPH 1,2) and two different positions within the crystallised area using the RCPL laser light (NGST RCPL 1,2) at R2 prism position for NGST film sample.

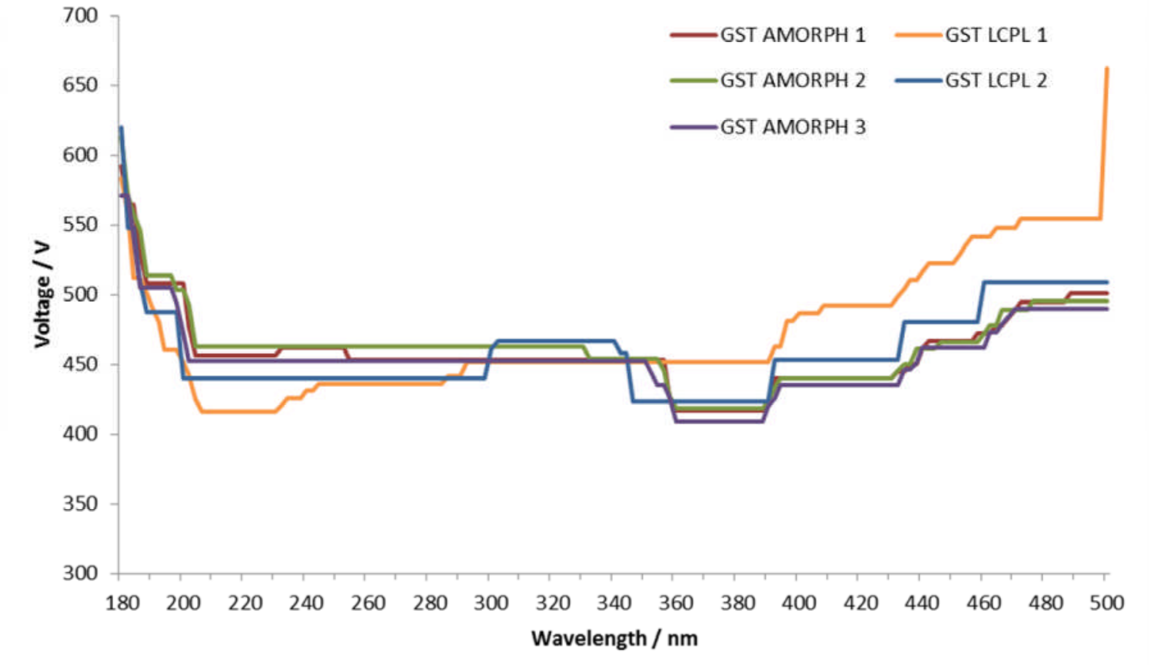

Figure S18. PMT voltages of spectral scans on three different amorphous regions (GST AMORPH 1,2,3) and two different positions within the crystallised area using the LCPL laser light (GST LCPL 1,2) at R1 prism position for GST film sample.

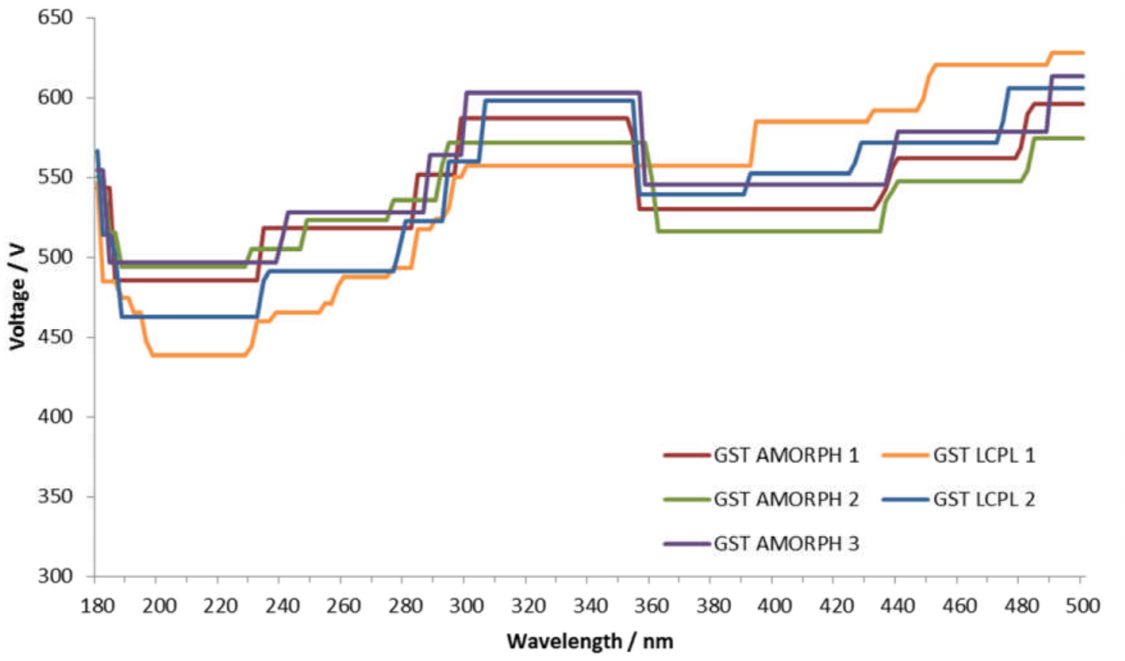

Figure S19. PMT voltages of spectral scans on three different amorphous regions (GST AMORPH 1,2,3) and two different positions within the crystallised area using the LCPL laser light (GST LCPL 1,2) at R2 prism position for GST film sample.

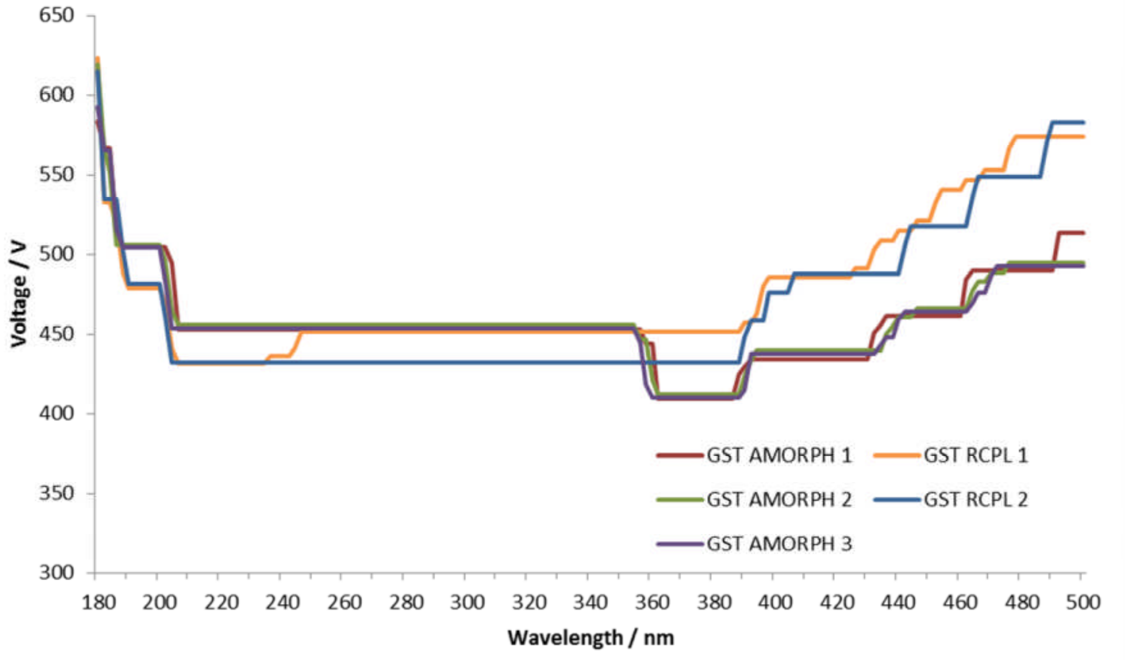

Figure S20. PMT voltages of spectral scans on three different amorphous regions (GST AMORPH 1,2,3) and two different positions within the crystallised area using the RCPL laser light (GST RCPL 1,2) at R1 prism position for GST film sample.

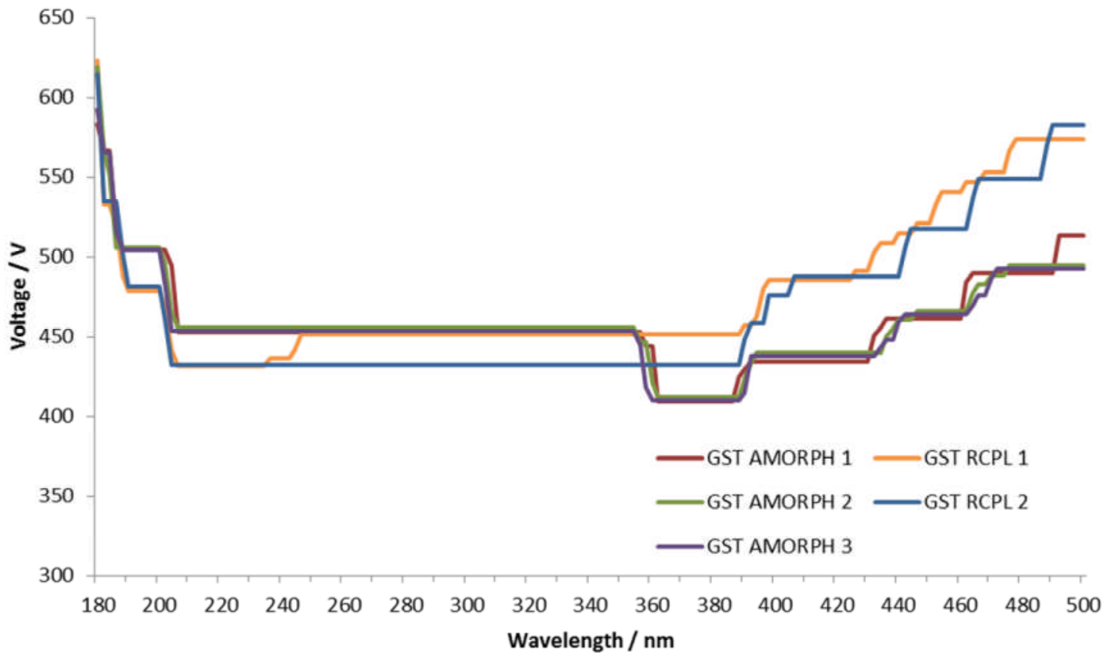

Figure S21. PMT voltages of spectral scans on three different amorphous regions (GST AMORPH 1,2,3) and two different positions within the crystallised area using the RCPL laser light (GST RCPL 1,2) at R2 prism position for GST film sample.

There is a clear difference between the CD spectra recorded from the crystallised areas induced by left and right circularly polarised light in the wavelength range between 380 to 420 nm for pure GST (Figure S24) and in a wider wavelength range from 410 to 500 nm for NGST (Figure S27). This difference between the L-CPL and R-CPL spectra is also noticeably larger for NGST. For both GST and NGST films, the spectra from the area crystallised by L-CPL have a consistently higher value of the CD than those from the area crystallised by R-CPL.

The spectra from NGST have pronounced mirror symmetry dependent on the handedness of the light used to induce crystallisation when compared to amorphous spectra wavelength range between 360-440 nm (Figure S27). A similar trend in the same spectral region is also observed for pure GST films but the changes are for the most part within the experimental standard deviations (Figure S24).

Some deviation from complete mirror symmetry in these spectra may be a consequence of the increasing contribution from linear dichroism and different degree

of crystallisation induced by the laser light illumination. In case of GST, the R-CPL causes larger detectable changes than the L-CPL light at about 500 nm, whereas in the case of NGST films, the change induced by L-CPL is considerably larger than the change caused by crystallisation using R-CPL in the wavelength range between 410 to 500 nm. Optical inspection and SEM images of the L-CPL and R-CPL crystallised spots on the NGST film indicate that the changes caused by light irradiation are more pronounced for the spot crystallised using L-CPL light.

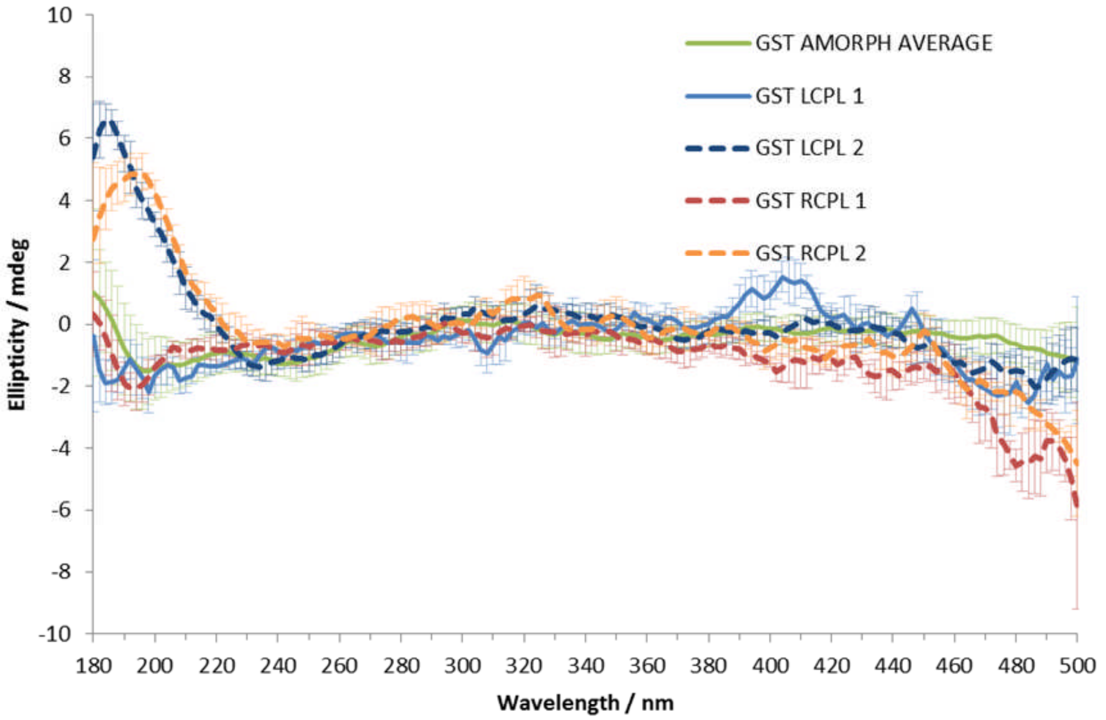

Figure S22. Comparison of averaged spectrum recorded from amorphous regions (GST AMORPH AVERAGE) with two individual spectra from two different positions within the crystallised areas induced by left (GST LCPL 1,2) and right (GST RCPL 1,2) circularly polarized laser light on GST films at the R1 prism position.

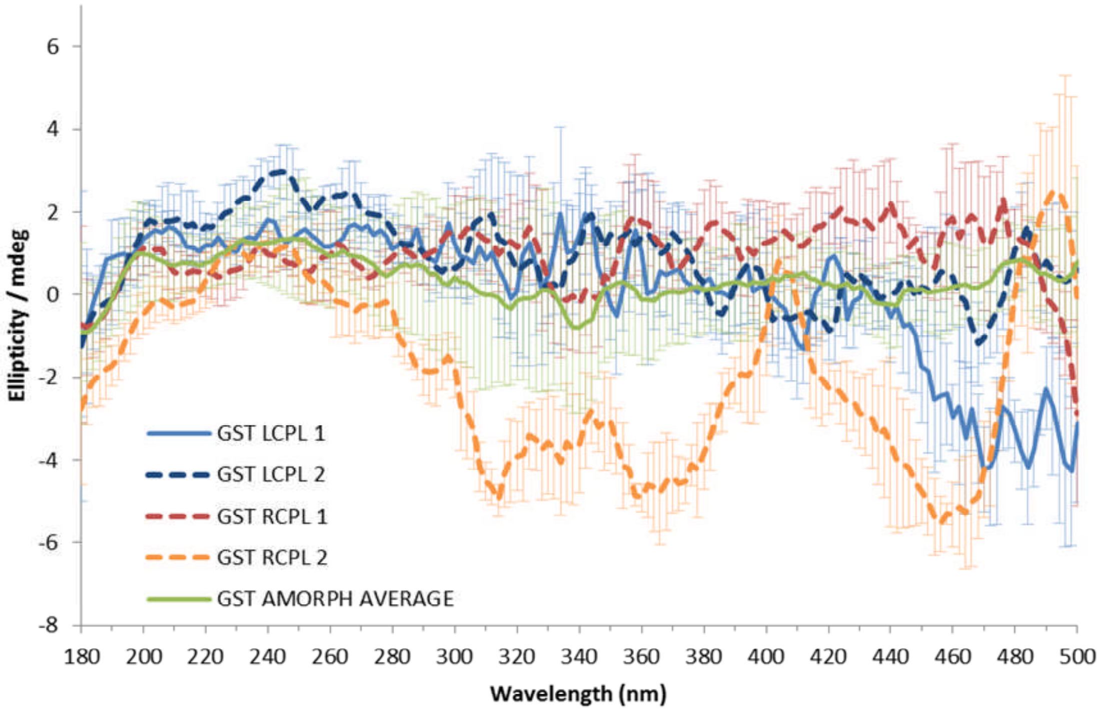

Figure S23. Comparison of averaged spectrum recorded from amorphous regions (GST AMORPH AVERAGE) with two individual spectra from two different positions within the crystallised areas induced by left (GST LCPL 1,2) and right (GST RCPL 1,2) circularly polarized laser light on GST films at the R2 prism position.

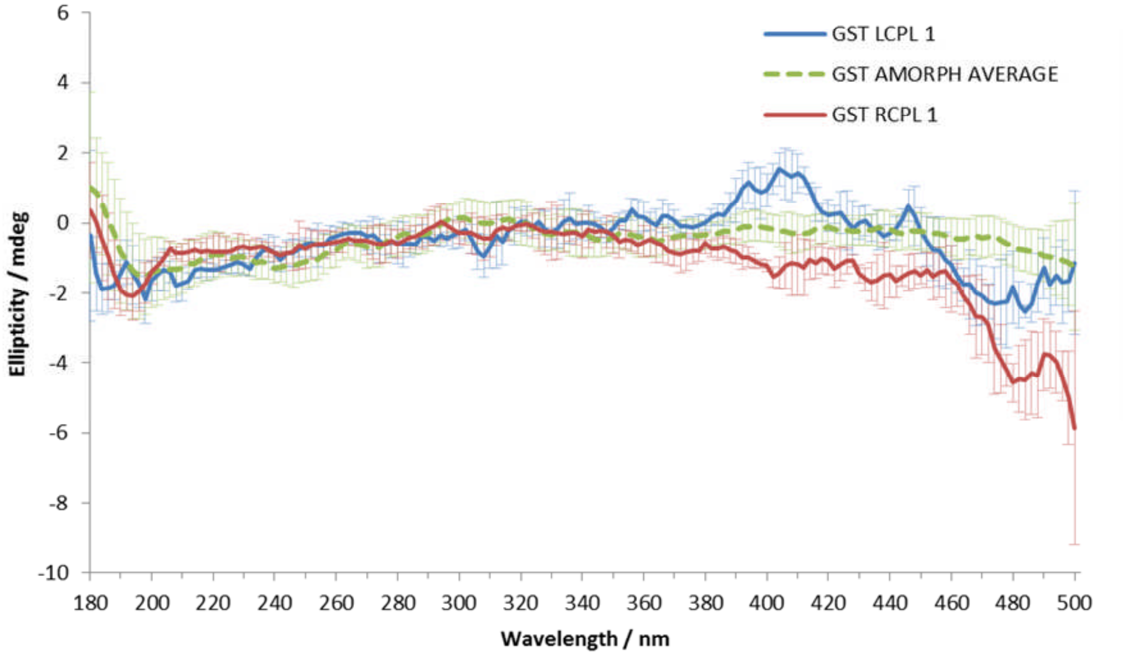

Figure S24. Comparison of averaged spectrum recorded from amorphous regions (GST AMORPH AVERAGE) with individual spectra from crystallised areas induced by left (GST LCPL 1) and right (GST RCPL 1) circularly polarized light on GST films at the R1 prism position.

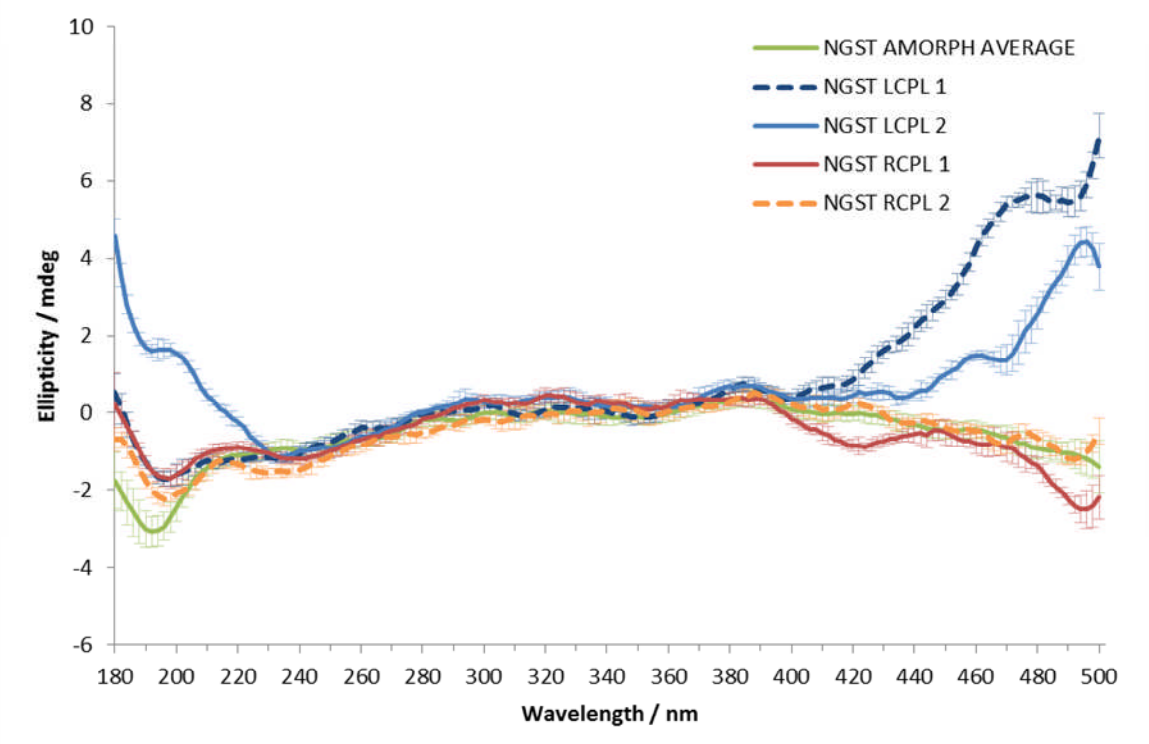

Figure S25. Comparison of averaged spectrum recorded from amorphous regions (NGST AMORPH AVERAGE) with two individual spectra from two different positions within the crystallised areas induced by left (NGST LCPL 1,2) and right (NGST RCPL 1,2) circularly polarized laser light on NGST films at the R1 prism position.

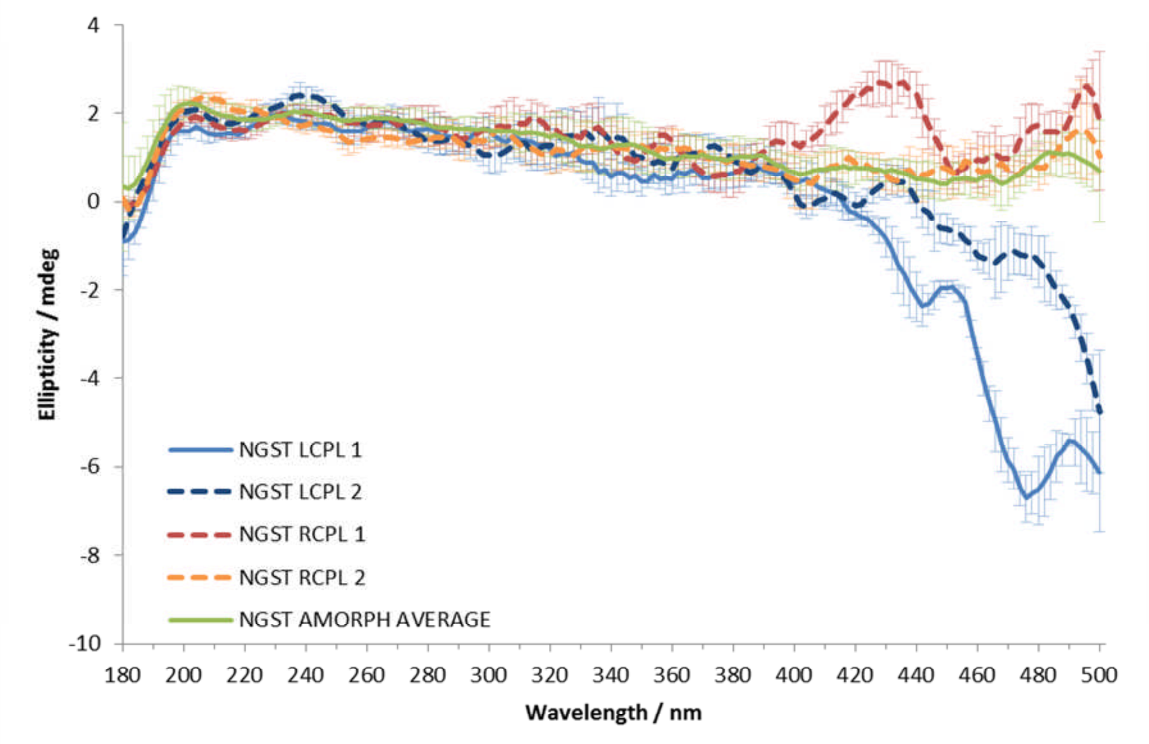

Figure S26. Comparison of averaged spectrum recorded from amorphous regions (NGST AMORPH AVERAGE) with two individual spectra from two different positions within the crystallised areas induced by left (NGST LCPL 1,2) and right

(NGST RCPL 1,2) circularly polarized laser light on NGST films at the R2 prism position.

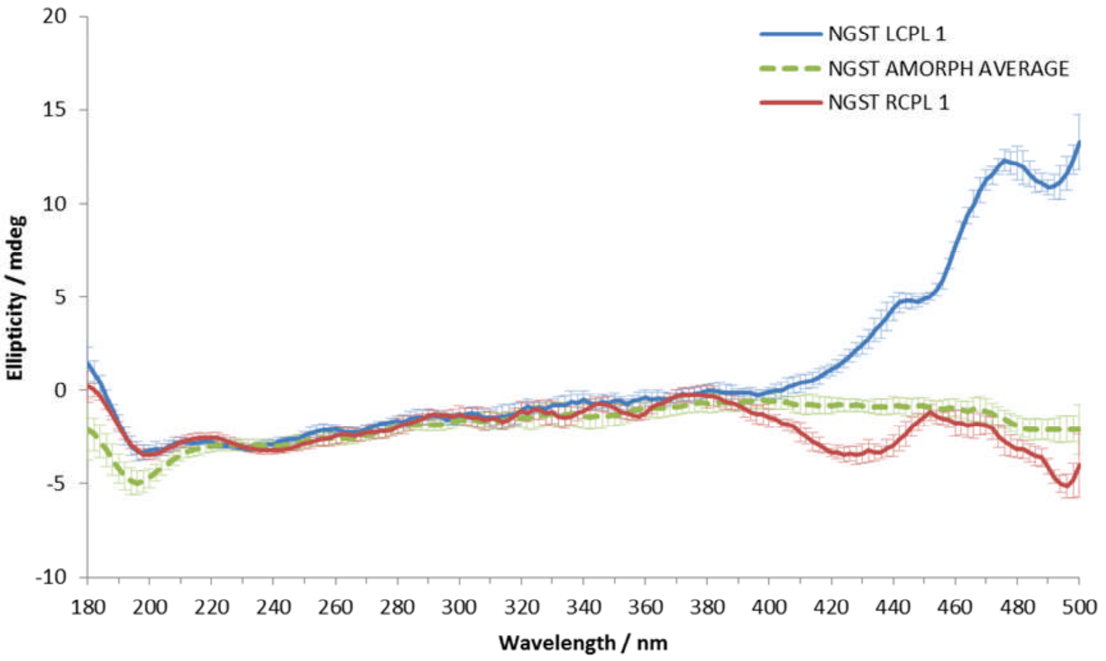

Figure S27. Comparison of double CD (R1-R2) averaged spectra recorded from amorphous regions (NGST AMORPH AVERAGE) with individual spectra from the crystallised areas induced by left (NGST LCPL 1) and right (NGST RCPL 1) circularly polarized laser light on NGST films.

The final spectra are examined in wavelength range between 360-440 nm for both films. Spectra recorded from the crystallised regions having higher PMT voltage values are used for the final comparison. The respective spectra are GST LCPL 1 and GST RCPL 1 for pure GST (Figure S24) and NGST LCPL 1 and NGST RCPL 1 for N-doped GST (Figure S27). In these figures the averaged spectra from the corresponding amorphous regions are also shown for reference.
